# Worldwide trends in the quality and breadth of clinical investigations of medical devices over the past decade: a scoping review and evidence map

**DOI:** 10.1186/s13063-025-08793-y

**Published:** 2025-03-29

**Authors:** David F. Keane, Cherian Mathew, Ryan Longley, Rosie Dunne, Thomas Mathew, Elaine Harris, Paddy Gillespie, Abhay Pandit, Matthew D. Griffin

**Affiliations:** 1 CÚRAM Research Ireland Centre for Medical Devices, University of Galway, Galway, Ireland; 2 Health Research Board Clinical Research Facility Galway, University of Galway, Galway, Ireland; 3 HRB Trials Methodology Research Network, University of Galway, Galway, Ireland; 4 Saolta University Hospital Group, Galway, Ireland; 5 Cardiac Physiology, Sherwood Forest Hospitals NHS Foundation Trust, Nottinghamshire, UK; 6 University of Galway Library, Galway, Ireland; 7 University of Limerick Hospital Group, Limerick, Ireland; 8 School of Chemical and Biopharmaceutical Sciences, Technological University Dublin, Dublin, Ireland; 9 Health Economics and Policy Analysis Centre, University of Galway, Galway, Ireland; 10 Regenerative Medicine Institute (REMEDI), School of Medicine, University of Galway, Galway, Ireland

**Keywords:** Medical device, Scoping review, Evidence map, Clinical trial

## Abstract

**Background:**

Recent regulatory developments in Europe have enhanced the requirements for clinical investigations of medical devices, partly in response to a perceived need for a higher level of evidence that is publicly available. This scoping review aims to map published clinical investigations of medical devices by device type and clinical specialty and summarise key trial design aspects.

**Methods:**

We developed a search for EMBASE that identified clinical investigations of medical devices during two discrete 3-month periods at the end of 2012 and of 2022. Core information from observational studies was extracted along with details on study design in interventional studies. We developed an evidence map of published studies across device type and clinical specialty and summarised study design aspects.

**Results:**

We included 682 studies from 2012 and 1682 studies from 2022. Around a quarter of studies were interventional and the remainder being observational and primary outcomes of effectiveness were more common than efficacy. Key study design aspects were frequently unreported, including sample size justification, registration, randomisation technique and funders. Our evidence map demonstrated that predominantly, investigations were of implants and were in a cardiovascular setting. Clinical investigations were broadly similar between 2012 and 2022, though there was a reduction in the proportion of cardiovascular studies, a move in the share of studies coming out of Europe and North America towards Asia and a general improvement in the quality of study design reported.

**Conclusions:**

Implanted devices in cardiovascular disease and orthopaedics are the focus of a large proportion of published clinical investigations of medical devices. Reporting of key study design aspects of clinical investigations of medical devices are improving but are still below expected requirements.

**Registration:**

A pre-specified and published protocol was registered on figshare: doi.org/10.6084/m9.figshare.22276945.v1 on 15/03/2023

**Supplementary Information:**

The online version contains supplementary material available at 10.1186/s13063-025-08793-y.

## Background

Evidence-based medicine is built upon clinical investigations which evaluate new drugs, devices or other interventions through a series of phased evaluations. Approval processes for new drugs usually require large-scale randomised controlled trials, but this has not historically been the case for medical devices [1, 2]. Key differences when evaluating devices include the relative importance of medical device user training and technique, iterative modifications to a device during development and the level of evidence required by regulators. This has led to medical devices being approved with little clinical data supporting effectiveness [3] and some concern about safety implications [4].

This historical dearth of clinical effectiveness evidence for medical devices has also had direct implications for value-based decision-making principles [5–7]. In particular, the framework of health technology assessment (HTA), adopted in many jurisdictions to inform reimbursement of and patient access to health technologies [8], is dependent on the availability of high-quality comparative effectiveness evidence [6]. In the absence of a formal HTA process, it becomes difficult to systematically assess if resources are being allocated efficiently or equitably.

Partly in response to these concerns, the European Union Medical Device Regulation (MDR) has introduced enhanced requirements for clinical evaluation of medical devices. This includes increasing transparency by requiring publication of clinical investigation reports, proactive post market surveillance, clinically meaningful comparator groups and clinically relevant endpoints [9]. In addition, guidance documents [10] and frameworks [11] have been developed to support planning and delivery of clinical studies. These enhanced regulatory standards give rise to the potential for better and more informed decision-making from both the clinical and cost effectiveness perspectives, but the extent to which the academic- and industry-based medical device communities have laid the groundwork to meet them is unclear.

Despite clear advocacy for improvements in the conduct and methodological rigour of trials, there has been very little detailed evaluation of the broad landscape of published clinical investigations of medical devices. We aimed, therefore, to comprehensively map published clinical investigations of medical devices across device type and clinical area, to synthesise key study design elements and to determine whether any notable changes had occurred in these areas over the last decade.

## Methods

### Study design

The study was informed by the Joanna Briggs Institute’s Manual for Evidence Synthesis [12] and The Preferred Reporting Items for Systematic Reviews and Meta-analyses extension for Scoping Reviews (PRISMA-ScR) [13] (checklist available in supplementary material (S1)). The pre-specified and published protocol for the study contains detailed methodology [14].

### Inclusion criteria

Participants: Studies must be clinical investigations carried out on human participants.

Concept: This review is focussed on aspects of study design.

Context: Studies must be original investigations of medical devices. We will work on the basis of the EU Medical Device Regulations definition [15] of a medical device but will exclude medical devices identified as:

- In vitro diagnostics
- Imaging devices that do not go on or in the human body (e.g. CT scanners)
- Software as a medical device
- Surgical instruments
- Devices used as part of dental procedures or for dental purposes

### Information sources and search strategy

We developed our search strategy in collaboration with an information specialist (RD). Only studies reporting primary research in the English language were included. The search was carried out in the EMBASE database. Grey literature and reference lists were not searched due to the number of studies identified in the primary search. The concepts to inform our search strategy development were based on our inclusion/exclusion criteria and the final search term can be seen in supplementary material (S2). To ensure screening and data extraction were feasible, we limited the review to studies that were entered into the database in the final quarter of 2022 (i.e. 01/10/2022 to 31/12/2022). To allow us to map changes over time, we repeated the search during the equivalent sampling period 10 years previously (01/10/2012 to 31/12/2012).

### Screening and study selection

All citations were imported into the Rayyan web-based software [16] for abstract screening. Two authors independently carried out abstract screening for the 2022 period (DK, RL) and for the 2012 period (DK, CM). Screening included assessment of the study against the inclusion criteria and further categorising the study as observational or interventional. Pilot testing of the screening process was carried out on 25 abstracts by all three screening authors together to agree on the process. All screening authors then screened a further 25 abstracts independently and full screening commenced on the basis that greater than 75% agreement was achieved. On screening completion, discrepancies were resolved by consensus review initially and with a third author where necessary (CM or RL).

### Data extraction

All studies that were categorised as observational during screening had study data extracted from the abstract only (full text review was not feasible) while for interventional studies, further information on study design and reporting was extracted from the full study report. Data extraction was split between three authors (DK, CM, TM). Twenty-five studies were extracted in duplicate and full extraction proceeded when agreement across categories exceeded 75%. Extraction categories and definitions can be found in supplementary material S3.

### Data synthesis

We used all studies to describe general characteristics of clinical investigations and only interventional studies to describe more detailed study design elements. All items extracted were tabulated for the two time periods investigated to illustrate any changes between 2012 and 2022. We used an evidence map to summarise primary study information (device type, clinical specialty, number of participants, study design) separately in 2012 and 2022. We demonstrated the global distribution of clinical investigations using choropleth maps for 2012 and 2022 based on the country of the corresponding author for each publication. Finally, we used Clarivate’s Journal Citation Reports to link the journal in which each article was published to an impact factor for years 2012 and 2022. Choropleth maps were generated using DataWrapper (https://www.datawrapper.de/) and charts generated using ‘R’ version 3.0.2 (R Foundation for Statistical Computing, Vienna, Austria).

### Patient and public involvement

Although there was no direct PPI in this paper, the research programme that funded this study was developed in collaboration with an embedded Public Engagement team (https://curamdevicesengage.ie/) who will support dissemination activities after publication.

## Results

### Search results

**Fig. 1:**
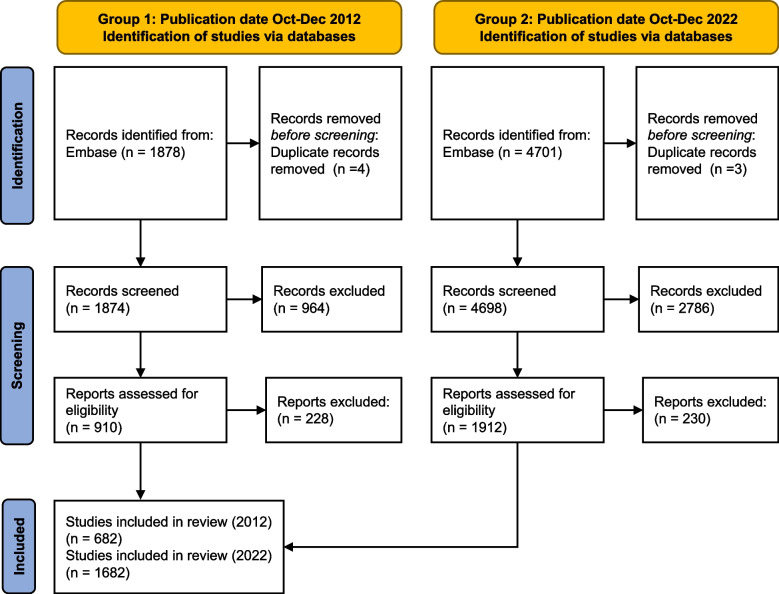
PRISMA flow diagram showing the numbers of studies identified, screened and included from the two discrete time periods, being the final 3 months of 2012 and 2022 as highlighted in the yellow boxes

After removing duplicates, our search identified 1874 studies in 2012 and 4698 studies in 2022 which were screened for inclusion. The inter-rater agreement between screeners at the abstract screening stage was 82%. In all cases, consensus was reached on whether an article should be rejected at the screening stage or not. In pilot testing for data extraction, inter-rater agreement was 90% (DK and CM) and 80% (DK and TC). After screening and assessment for eligibility, 682 and 1682 studies were included in the review, as described in the flow chart in Fig. 1.

### Trends over time

We found a 2.5-fold increase in the number of studies published during the sampling periods that fit our inclusion criteria between 2012 and 2022. The proportion of studies that was classified as interventional, however, was similar for the time periods (24% and 21%). In 2012, 66% of investigations originated in Europe and North America whereas this figure was only 57% in 2022. In contrast, the proportion conducted in Asia increased from 29 to 36%. This pattern was consistent for both interventional and observational studies. Cardiovascular investigations also had a relative reduction over the last decade, from 29 to 24% of all studies.

There was evidence of improvement in a number of indicators of the quality of reporting of published studies from 2012 to 2022. This included increases in the proportions of studies that detailed ethical or institutional review board approval (from 89 to 97%), that were accompanied by publicly available trial registration (16 to 53%) and that included a justification of sample size (31 to 47%). There were also increases in the proportion of studies reporting randomisation approaches (44 to 54%) and blinding (27 to 30%) also increased, along with a modest increase in the proportion using placebo or sham devices as comparators (4 to 7%). The proportion of publications that did not make a declaration in relation to funding of the investigation reduced, though was still high at 20% in 2022. Industry funded studies made up a largely unchanged share of just over 20%. Fewer studies in 2022 were published in journals without an impact factor (from 15 to 11%).

### Study design elements of observational studies

Observational studies made up over three quarters of published studies across the two sampling periods. Prospectively designed data collection was evident in registry studies (4%), diagnostic testing studies (4%) and other prospective observational studies (21%) but almost three quarters were case studies or retrospective studies. Reflecting the very different study types, there was a wide spread of the numbers of recruited participants from under 10 to above 1000. Primary outcomes tended to focus more on effectiveness than efficacy and nearly a third had a safety focussed primary outcome (Table 1).

**Table 1:** Characteristics of included studies

	**2012 (*n* = 682)**		**2022 (*n* = 1682)**	
	**Observational** ***n* = 518 (76%)**	**Interventional** ***n* = 164 (24%)**	**Observational** ***n* = 1334 (79%)**	**Interventional** ***n* = 348 (21%)**
**Study type**				
Case study/series	163 (31%)	–	355 (27%)	–
Retrospective	220 (42%)	–	586 (44%)	–
Prospective observational	65 (13%)	–	281 (21%)	–
Registry	44 (8%)	–	55 (4%)	–
Diagnostic testing	26 (5%)	–	57 (4%)	–
Single arm trial	–	27 (16%)	–	62 (18%)
Controlled trial	–	25 (15%)	–	40 (11%)
Randomised controlled trial	–	112 (68%)	–	246 (71%)
**Number of participants**				
< 10	147 (28%)	6 (4%)	349 (26%)	17 (5%)
10–49	135 (26%)	72 (44%)	321 (24%)	136 (39%)
50–99	69 (13%)	40 (24%)	211 (16%)	87 (25%)
100–499	113 (22%)	42 (26%)	308 (23%)	95 (27%)
500–999	17 (3%)	3 (2%)	46 (3%)	7 (2%)
≥ 1000	37 (7%)	1 (1%)	99 (7%)	6 (2%)
**Device type**				
Active implant	46 (9%)	6 (4%)	155 (12%)	6 (2%)
Implant	207 (40%)	33 (20%)	542 (41%)	59 (17%)
Stent	62 (12%)	17 (10%)	146 (11%)	22 (6%)
Artificial organ	12 (2%)	15 (9%)	27 (2%)	49 (14%)
Catheter	4 (1%)	4 (2%)	56 (4%)	2 (1%)
Diagnostic	49 (9%)	11 (7%)	121 (9%)	16 (5%)
Dressing/bandage	3 (1%)	8 (5%)	15 (1%)	20 (6%)
Drug delivery	13 (3%)	10 (6%)	55 (4%)	26 (7%)
External prosthetic	58 (11%)	18 (11%)	64 (5%)	67 (19%)
Energy/stimulation	64 (12%)	42 (25%)	153 (11%)	81 (23%)
**Clinical specialty**				
Anaesthesia/respiratory/critical care	22 (4%)	24 (15%)	65 (5%)	58 (17%)
Cardiovascular	161 (31%)	34 (21%)	372 (28%)	37 (11%)
Dermatology/wounds/plastics	36 (7%)	20 (12%)	73 (5%)	43 (12%)
Diabetes/nutrition	10 (2%)	2 (1%)	29 (2%)	14 (4%)
Internal medicine	13 (3%)	11 (7%)	88 (7%)	18 (5%)
Neurology/ophthalmology/audiology	71 (14%)	17 (10%)	210 (16%)	43 (12%)
Oncology/immunology	22 (4%)	5 (3%)	41 (3%)	10 (3%)
Orthopaedics/rehabilitation	121 (23%)	29 (18%)	269 (20%)	82 (24%)
Paediatrics	29 (6%)	12 (7%)	97 (7%)	19 (5%)
OB/GYN/urology/sexual health	33 (6%)	10 (6%)	90 (7%)	24 (7%)
**Primary outcome**				
Safety	85 (16%)	5 (3%)	222 (17%)	2 (1%)
Efficacy	47 (9%)	74 (45%)	169 (13%)	130 (37%)
Safety and efficacy	72 (14%)	24 (15%)	25 (2%)	26 (7%)
Effectiveness	231 (45%)	55 (34%)	723 (54%)	132 (38%)
Safety and effectiveness	58 (11%)	6 (4%)	131 (10%)	34 (10%)
Diagnostic testing	25 (5%)	0 (0%)	64 (5%)	0 (0%)
Pilot/feasibility	–		–	24 (7%)
**Study region**				
Africa	3 (1%)	6 (4%)	18 (1%)	13 (4%)
Asia	143 (28%)	56 (34%)	471 (35%)	142 (41%)
Australia	5 (1%)	5 (3%)	31 (2%)	17 (5%)
Europe	192 (37%)	59 (36%)	469 (35%)	102 (29%)
North America	161 (31%)	36 (22%)	328 (25%)	64 (18%)
South America	14 (3%)	2 (1%)	17 (1%)	10 (3%)
**Impact factor**				
% with no impact factor	83 (16%)	19 (12%)	155 (12%)	28 (8%)
Median	2.3	2.5	2.6	3
Maximum	38.9	51.7	169	159

### Study design elements of interventional studies

The majority of interventional studies across the two sampling periods were single centre randomised controlled trials (RCTs) assessing a device against a competitor device or against standard care. Only 7% of interventional studies incorporated a placebo or sham as comparator (Table 2). In 2022, funding was most likely to be academic/governmental with 22% of studies declaring industry funding. Lack of methodological rigour was evident in a substantial proportion of studies, with no documentation of sample size calculations in 53% and of trial registration in 47% of studies and funding details not declared in 30%. For most studies, randomisation was simple randomisation with a computer-generated sequence and no allocation concealment or blinding. Primary outcomes were again more focussed on effectiveness than efficacy, though safety outcomes were less commonly investigated than in observational studies (18% as compared to 28% in 2022).

**Table 2:** Study design aspects of interventional studies

	**2012 (*n* = 164)**	**2022 (*n* = 348)**
**Number of sites**		
1	134 (82%)	304 (87%)
2–10	17 (10%)	21 (6%)
> 10	13 (8%)	23 (7%)
**Ethics/IRB approval detailed**		
Yes	147 (89%)	336 (97%)
No	17 (10%)	12 (3%)
**Sample size justified**		
Yes	51 (31%)	152 (47%)
No	113 (69%)	174 (53%)
**Trial registration detailed**		
Yes	27 (16%)	185 (53%)
No	137 (84%)	163 (47%)
**Funding**		
Not detailed	81 (49%)	103 (30%)
Declaration of no funding	17 (10%)	69 (20%)
Academic/government/charity	29 (16%)	101 (29%)
Industry	37 (21%)	75 (22%)
**Comparator**		
* Number of studies with comparator*	*N* = *137 (84%)*	*N* = *287 (82%)*
Placebo/sham	5 (4%)	19 (7%)
Standard care	50 (36%)	136 (47%)
Competitor device	82 (60%)	131 (46%)
**Randomisation and blinding**		
* Number of randomised studies*	*N* = *112*	*N* = *286*
*Randomisation technique*		
Not detailed	43 (38%)	111 (39%)
Simple	55 (49%)	117 (41%)
Block	7 (6%)	38 (13%)
Stratified	7 (6%)	17 (6%)
Minimised		3 (1%)
*Random allocation generation*		
Not detailed	63 (56%)	131 (46%)
Computer generated	35 (31%)	124 (43%)
Random number table/list	6 (4%)	19 (7%)
Casting lots	4 (4%)	6 (2%)
Quasi-randomisation	4 (4%)	6 (2%)
*Allocation concealment*		
Not detailed	73 (65%)	209 (73%)
Sealed envelopes	31 (28%)	52 (18%)
Independent central allocation	8 (7%)	25 (9%)
*Blinding*		
No blinding/not detailed	71 (63%)	171 (60%)
Patient	13 (11%)	68 (24%)
Observer/outcome assessor	33 (29%)	88 (31%)

### Evidence map of clinical investigations

**Fig. 2:**
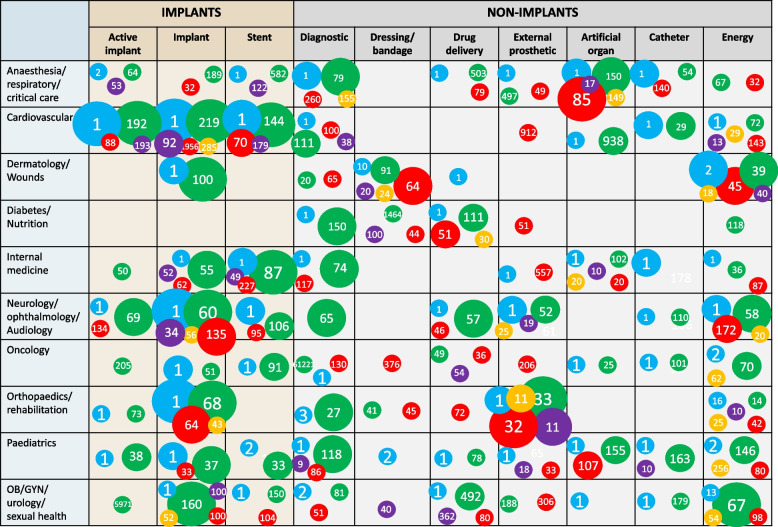
Evidence map detailing key study design aspects by clinical specialty and device type. Size of circle: number of studies (1–5; 6–10; 11–20; 21 +). Number in circle: median number of participants. Circle colour: blue = case study/series; green = observational; purple = single arm trial; orange = non-randomised comparative study; red = randomised controlled trial

The evidence map for studies from 2022 can be seen in Fig. 2 and the corresponding map for 2012 can be seen in supplementary material (S4). Implantable devices (implant, active implant and stent categories) represented a large proportion of investigations. Despite the huge burden of diabetes and cancer globally, there were relatively few investigations in these areas with cardiovascular and orthopaedics/rehabilitation having the greatest share of studies.

Notable differences were evident in the number of studies providing the highest grade of evidence. For example, there were relatively few interventional studies (yellow, purple, red circles) in diagnostics, active implants, catheters or stents and also for cardiovascular and paediatrics clinical specialties. Conversely, investigations in drug delivery and artificial organs and in anaesthesia/respiratory/critical care were relatively well served by interventional studies. As would be expected, observational studies had a higher participant recruitment than other study designs.

### Country of origin of clinical investigations

**Fig. 3:**
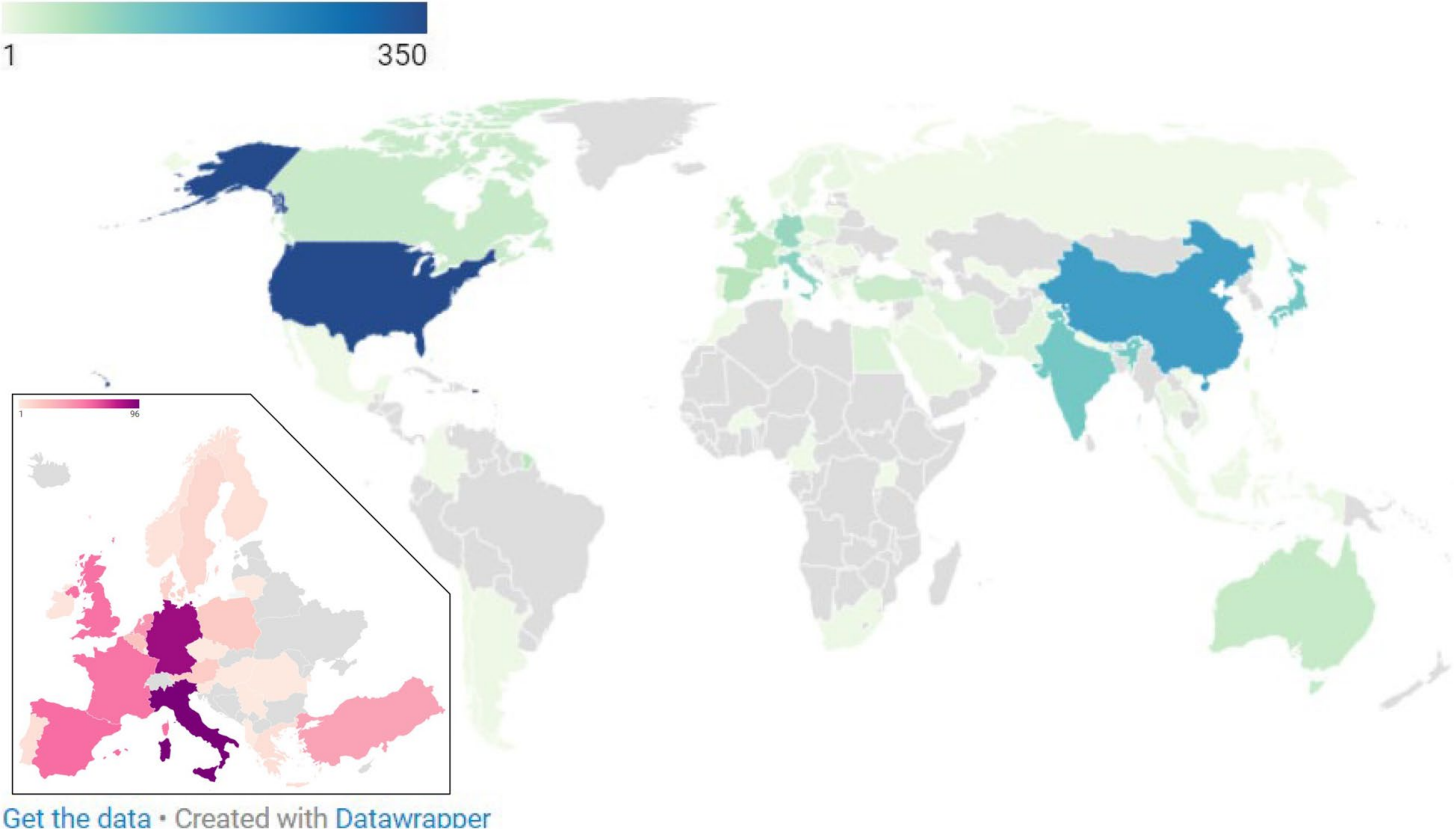
Choropleth map of the countries associated with the corresponding author of all studies. Inset map of Europe scaled to total studies in Europe only

In 2022, more than twice as many clinical medical device studies were associated with the USA than with any other country. China, India and Japan were associated with the next highest numbers of studies (Fig. 3). For the same time period within Europe, the highest numbers of studies were associated with Italy (96 studies) and Germany (87 studies). When both time periods were considered and both observational and interventional studies were included, these two countries were joined with France, Spain, UK and Turkey as generating the highest number of studies. Studies originating in Africa and Asia were more likely to be interventional than observational while those in Europe were more likely to be observational.

### Journal impact factor of clinical investigations

**Fig. 4:**
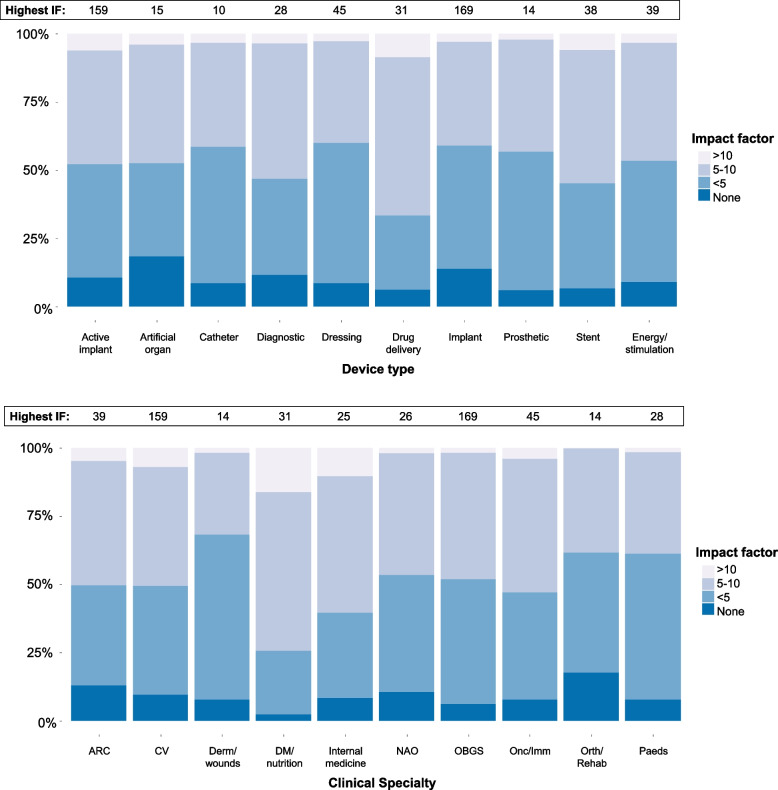
Impact factors (IF) of the journals that the clinical investigations were published in, by clinical specialty and by device type. ARC: anaesthesia, respiratory, critical care; CV: cardiovascular; Derm/wounds: dermatology, wound healing; DM: diabetes mellitus; NAO: neurology, audiology, ophthalmology; Onc/imm: oncology, immunology; Orth/rehab: orthopaedics, rehabilitation; Paeds: paediatrics

In 2022, 89% of all articles were published in journals with an impact factor, ranging from 79% for case studies to 98% of single arm trials (SATs). The impact factors by clinical area and device type are shown in Fig. 4. Articles published in journals with the highest impact factors were a SAT of an implantable cardioverter defibrillator (impact factor 159), a RCT of a cerebral embolic protection device (impact factor of 159) and a retrospective study of the safety of an intrauterine device (impact factor 169).

## Discussion

This scoping review of clinical investigations of medical devices demonstrates increases in the number and quality of published clinical investigations from 2012 to 2022, albeit best practice continued to be lacking in some key methodological components. Implanted devices—particularly in orthopaedics and cardiology—contribute the largest share of published studies, while there were relatively few studies in diabetes/nutrition, oncology and paediatrics. Our results also highlight a geographical shift, with a higher proportion of studies being conducted in Asia and lower proportions in Europe and North America in 2022 compared to 2012.

The total number of citation results in Medline increased by a factor of 1.2 between 2012 and 2022, yet our search results showed a 2.5-fold increase in the number of investigations fitting our inclusion criteria. This is consistent with a review of interventional studies registered on ClinicalTrials.gov, which showed a relative reduction in drug trials and an increase in medical device trials from 4.2 to 14.7% between 2000 and 2020 [17]. As both the FDA [18] and regulators in the EU [19] have highlighted the need for clinical data to support medical devices, this can be considered as an encouraging finding, though we cannot necessarily infer that the clinical data supporting any given device has increased as there has also been an increase in the number of devices submitted for approval [20]. There was a small reduction in the proportion of studies that were interventional and in the proportion that were RCTs (from 16 to 15%), suggesting that although the number of interventional studies of devices has increased, observational studies also continue to rise.

Previous reviews of the type and quality of published evidence of medical devices have looked at high-risk cardiovascular [21], orthopaedic [22] and paediatric [23] devices approved in the EU and cardiovascular devices approved by the FDA in the USA [24]. These reviews consistently describe a picture of inadequate strength of evidence, including low numbers of randomised studies and control groups, lack of blinding, small sample sizes and a lack of sample size justification. Each of these issues is reinforced by the findings of our review, despite the encouraging trends over time. Specific challenges to medical device trials along with potential solutions have previously been well discussed [25, 26]. Further opportunities are now being provided by developments in trial design, including pragmatic and adaptive designs, Bayesian approaches and integration of human factors into study design [27]. Furthermore, regulators across the world, including in the USA, the EU, the UK and China, are increasingly open to real-world data (RWD) and real-world evidence (RWE) and are producing frameworks and guidance to facilitate this [28]. Development of computing infrastructure, trusted research environments (TREs) and ability to link datasets provides huge potential for efficient studies with large numbers of participants.

We observed a notable shift away from Europe and North America towards proportionately more trials being carried out in Asia. This trend has been well signposted for Europe. Recent surveys of device manufacturers about the implications of the MDR found that 50% were deprioritising the EU market [29] and that some were planning to remove devices from the EU market on expiration of their certificate. However, our data suggesting a relative reduction in studies coming out of the USA is more unexpected. Generally, there is a perception that accelerated pathways in the USA (such as 510(k)) and the negative effects of the MDR have made the USA more favourable than the EU for launching novel devices [30]. The growth in studies in Asia has previously been shown for pharmaceutical studies and artificial intelligence enabled devices and is likely multifactorial in nature, including rapid economic growth and regulatory reform [31, 32].

Our evidence map showed that much of the published evidence was for implantable medical devices, though this was primarily due to observational studies. In fact, among interventional studies, non-implantable devices were more common. Given that most active implantable devices would have risk class III, it is notable how few of these studies were interventional. A World Health Organisation report on clinical evidence for medical devices called for high-quality randomised trials, wherever possible, before high-risk medical devices are approved to the market [33]. From a clinical perspective, the highest proportions of devices were orthopaedic and cardiovascular, reflecting the dominance of implanted devices in these areas, and it was notable that there were few studies in paediatrics, diabetes/nutrition and oncology. Our data support previous literature reviews showing much fewer RCTs of cardiovascular implants than of orthopaedic implants [21, 22]. Our results also validate a recent evaluation of paediatric studies which highlighted small numbers of participants and a lack of control group in paediatric trials of devices [23] while revealing relatively few comparative studies when compared to non-paediatric trials.

Strengths of this study include a well-developed search strategy to enable the review of such a broad topic, independent screening by two reviewers and assessment of reproducibility of data extraction between reviewers. The scoping review by nature was not exhaustive and, given the breadth of the scope, does not provide absolute information on the degree of evidence for a particular device or clinical area, risk class of device or whether devices under investigation had been approved for clinical use.

## Conclusion

Considering the positive trend in the availability of high-quality clinical effectiveness evidence for medical devices provides encouragement to ongoing efforts to elevate and expand the role of evidence-based decision-making in the context of medical devices technologies. Nevertheless, it is clear that there have been persistent gaps in the type and quality of evidence being captured by recent clinical studies of medical devices. This suggests the need for more robust incorporation of clinical trial design methodology and expertise into the earlier stages of medical device development. This would increase the yield of high-quality evidence to underpin patient safety and enable wider adoption of value-based principles and better-informed decision-making from both clinical and cost effectiveness perspectives.

## Supplementary Information

Supplementary Material 1.

## Data Availability

Summary data are presented in the manuscript and appendix. Further data that supports the findings of this study are available from the corresponding authors upon reasonable request.

## References

[1] Turner JR, Hoofwijk TJ. Clinical trials in new drug development. J Clin Hypertens (Greenwich). 2013;15(5):306–9. 10.1111/jch.12085. 23614843 10.1111/jch.12085 PMC8033831

[2] Marcus HJ, Payne CJ, Hughes-Hallett A, et al. Regulatory approval of new medical devices: cross sectional study. BMJ. 2016;20(353):i2587. 10.1136/bmj.i2587. 10.1136/bmj.i2587 PMC4875244 27207165

[3] Zuckerman DM, Brown P, Nissen SE. Medical device recalls and the FDA approval process. Arch Intern Med. 2011;171(11):1006–11. 10.1001/archinternmed.2011.30. 21321283 10.1001/archinternmed.2011.30

[4] Heneghan C, Thompson M, Billingsley M, et al. Medical-device recalls in the UK and the device-regulation process: retrospective review of safety notices and alerts. BMJ Open. 2011;1(1):e000155. 10.1136/bmjopen-2011-000155. 22021778 10.1136/bmjopen-2011-000155 PMC3191575

[5] Ming J, He Y, Yang Y, et al. Health technology assessment of medical devices: current landscape, challenges, and a way forward. Cost Eff Resour Alloc. 2022;20(1):54. 10.1186/s12962-022-00389-6. 10.1186/s12962-022-00389-6 PMC9533595 36199144

[6] Tarricone R, Torbica A, Drummond M. MedTecHTA project group. Health Econ. 2017;26 Suppl 1:145–52. 10.1002/hec.3468. 28139086 10.1002/hec.3468

[7] Torbica A, Tarricone R, Schreyögg J, Drummond M. Pushing the boundaries of evaluation, diffusion, and use of medical devices in Europe: insights from the COMED project. Health Econ. 2022S;31 Suppl 1:1–9. 10.1002/hec.4600. 36068719 10.1002/hec.4600

[8] Neumann P, Sanders G, Russell L, Siegel J, Ganiats T. Cost-effectiveness in health and medicine. New York, USA: Oxford University Press; 2017.

[9] Hoogervorst LA, Geurkink TH, Lübbeke A, et al. Quality and utility of European cardiovascular and orthopaedic registries for the regulatory evaluation of medical device safety and performance across the implant lifecycle: a systematic review. Int J Health Policy Manag. 2023;12: 7648. 10.34172/ijhpm.2023.7648. 37579359 10.34172/ijhpm.2023.7648 PMC10702370

[10] Bernard A, Vaneau M, Fournel I, Galmiche H, Nony P, Dubernard JM. Methodological choices for the clinical development of medical devices. Med Devices (Auckl). 2014;23(7):325–34. 10.2147/MDER.S63869. 10.2147/MDER.S63869 PMC4181748 25285025

[11] Sedrakyan A, Campbell B, Merino JG, et al. IDEAL-D: a rational framework for evaluating and regulating the use of medical devices. BMJ. 2016;353: i2372. 27283585 10.1136/bmj.i2372

[12] Peters MDJ, Godfrey C, McInerney P, Munn Z, Tricco AC, Khalil H. Chapter 11: scoping reviews (2020 version). In: Aromataris E, Mnn Z, editors. JBI manual for evidence synthesis. Adelaid: JBI; 2020.

[13] Tricco AC, Lillie E, Zarin W, O’Brien KK, Colquhoun H, Levac D, et al. PRISMA Extension for Scoping Reviews (PRISMA-ScR): checklist and explanation. Ann Intern Med. 2018;169(7):467–73. 30178033 10.7326/M18-0850

[14] Keane D, Study design of clinical investigations of medical devices: protocol for a scoping review and evidence map. figshare. 2023. Preprint. https://doi.org/10.6084/m9.figshare.22276945.v1.

[15] European Union Medical devices regulation (EU) 2017/745. 2017. https://eur-lex.europa.eu/legal-content/IT/ALL/?uri=celex:32017R0745. Accessed 01/03/24.

[16] Ouzzani M, Hammady H, Fedorowicz Z, Elmagarmid A. Rayyan — a web and mobile app for systematic reviews. Syst Rev. 2016;5:210. 10.1186/s13643-016-0384-4. 27919275 10.1186/s13643-016-0384-4 PMC5139140

[17] Gresham G, Meinert JL, Gresham AG, Piantadosi S, Meinert CL. Update on the clinical trial landscape: analysis of ClinicalTrials.gov registration data, 2000-2020. Trials. 2022;23(1):858. 10.1186/s13063-022-06569-2. 10.1186/s13063-022-06569-2 PMC9540299 36203212

[18] US Food and Drug Administration. Recommendations for the use of clinical data in premarket notification [510(k)] submissions. Draft guidance. 2023. https://www.fda.gov/regulatory-information/search-fda-guidance-documents/recommendations-use-clinical-data-premarket-notification-510k-submissions.

[19] Kearney B, McDermott O. The challenges for manufacturers of the increased clinical evaluation in the European medical device regulations: a quantitative study. 2023. Ther Innov Regul Sci. 2023;57:783–96. 10.1007/s43441-023-00527-z. 37198369 10.1007/s43441-023-00527-z PMC10276779

[20] Shuren J. Reflections on a record year for novel device innovation despite COVID-19 challenges https://www.fda.gov/news-events/fda-voices/reflections-record-year-novel-device-innovation-despite-covid-19-challenges. Accessed 01/03/24.

[21] Siontis GCM, Coles B, Häner JD, et al. Quality and transparency of evidence for implantable cardiovascular medical devices assessed by the CORE-MD consortium. Eur Heart J. 2024;45(3):161–77. 10.1093/eurheartj/ehad567. 37638967 10.1093/eurheartj/ehad567

[22] Lübbeke A, Combescure C, Barea C, et al. Clinical investigations to evaluate high-risk orthopaedic devices: a systematic review of the peer-reviewed medical literature. EFORT Open Rev. 2023;8(11):781–91. 10.1530/EOR-23-0024. 37909694 10.1530/EOR-23-0024 PMC10646516

[23] Guerlich K, Patro-Golab B, Dworakowski P, et al. Evidence from clinical trials on high-risk medical devices in children: a scoping review. Pediatr Res. 2024;95(3):615–24. 10.1038/s41390-023-02819-4. 37758865 10.1038/s41390-023-02819-4 PMC10899114

[24] Dhruva SS, Bero LA, Redberg RF. Strength of study evidence examined by the FDA in premarket approval of cardiovascular devices. JAMA. 2009;302(24):2679–85. 20040556 10.1001/jama.2009.1899

[25] Fox-Rawlings SR, Gottschalk LB, Doamekpor LA, Zuckerman DM. Diversity in medical device clinical trials: do we know what works for which patients? Milbank Q. 2018;96(3):499–529. 10.1111/1468-0009.12344. 30203600 10.1111/1468-0009.12344 PMC6131322

[26] Neugebauer EAM, Rath A, Antoine SL, et al. Specific barriers to the conduct of randomised clinical trials on medical devices. Trials. 2017;18(1):427. 28903769 10.1186/s13063-017-2168-0 PMC5597993

[27] Pazart L, Pelayo S, Chevallier T, et al. Threats and opportunities for the clinical investigation of high-risk medical devices in the context of the new European regulations. In: 14th International Joint Conference on Biomedical Engineering Systems and Technologies - BIOSTEC 2021. Special session on dealing with the change in European Regulations for Medical Devices. Vienna; 2021. p. 274-284. 10.5220/0010382902740284.

[28] Burns L, Le Roux N, Kalesnik-Orszulak R, et al. Real-world evidence for regulatory decision-making: updated guidance from around the world. Front Med (Lausanne). 2023;30(10):1236462. 10.3389/fmed.2023.1236462. 10.3389/fmed.2023.1236462 PMC10643567 38020096

[29] Kearney B, McDermott O. Challenges faced by manufacturers with clinical evaluation under the new European medical device regulations. Cogent Engineering. 2023;10:2. 10.1080/23311916.2023.2261236.

[30] Tarricone R, Banks H, Ciani O, et al. An accelerated access pathway for innovative high-risk medical devices under the new European Union medical devices and health technology assessment regulations? Analysis and recommendations. Expert Rev Med Devices. 2023;20(4):259–71. 10.1080/17434440.2023.2192868. 36987818 10.1080/17434440.2023.2192868

[31] Wu YH, Li FA, Fan YT, Tu PW. A study of medical device regulation management model in Asia. Expert Rev Med Devices. 2016;13(6):533–43. 10.1080/17434440.2016.1184970. 27136699 10.1080/17434440.2016.1184970

[32] Serra-Burriel M, Locher L, Vokinger KN. Development pipeline and geographic representation of trials for artificial intelligence/machine learning–enabled medical devices (2010 to 2023). NEJM AI. 2023;1(1). 10.1056/AIpc2300038.

[33] World Health Organisation. Clinical evidence for medical devices: regulatory processes focussing on Europe and the United States of America. Background paper 3. Medical devices: managing the mismatch. An outcome of the Priority Medical Devices project. Geneva: WHO; 2010.

